# Cellular Signaling at the Nano-Bio Interface: Spotlighting Membrane Curvature

**DOI:** 10.1146/annurev-physchem-090722-021151

**Published:** 2025-04

**Authors:** Chih-Hao Lu, Christina E. Lee, Melissa L. Nakamoto, Bianxiao Cui

**Affiliations:** 1 Department of Chemistry, Stanford University, Stanford, California, USA;; 2 Wu-Tsai Neuroscience Institute and Sarafan ChEM-H Institute, Stanford University, Stanford, California, USA; 3 Biophysics Program, Stanford University School of Medicine, Stanford, California, USA

**Keywords:** nano-bio interface, surface nanotopography, membrane curvature

## Abstract

No longer viewed as a passive consequence of cellular activities, membrane curvature—the physical shape of the cell membrane—is now recognized as an active constituent of biological processes. Nanoscale topographies on extracellular matrices or substrate surfaces impart well-defined membrane curvatures on the plasma membrane. This review examines biological events occurring at the nano-bio interface, the physical interface between the cell membrane and surface nanotopography, which activates intracellular signaling by recruiting curvature-sensing proteins. We encompass a wide range of biological processes at the nano-bio interface, including cell adhesion, endocytosis, glycocalyx redistribution, regulation of mechanosensitive ion channels, cell migration, and differentiation. Despite the diversity of processes, we call attention to the critical role of membrane curvature in each process. We particularly highlight studies that elucidate molecular mechanisms involving curvature-sensing proteins with the hope of providing comprehensive insights into this rapidly advancing area of research.

## INTRODUCTION

The nano-bio interface, which refers to the physical interface between the cell membrane and nanoscale extracellular surface topography, is important in a range of applications, including medical/dental implants (1, 2), biomedical nanotechnology (3), cell adhesions (4, 5), cell-extracellular matrix (ECM) interactions (6), and drug delivery (7, 8). Today, as biological and medical technologies move toward the nanoscale, understanding interactions at the cell-material interface and how they depend on surface topography is of increasing importance. Cells intimately interact with extracellular materials they come into contact with. The physical and mechanical properties of substrates such as surface topographical features, stiffness, and geometry greatly impact downstream effects, such as cell mechanics and signaling (9, 10).

Studies have shown that surface topography induces local plasma membrane curvatures, which then serve as mechanical and biochemical signals for further intracellular signaling transduction (11). For instance, surface protrusions imprint plasma membrane invaginations, which recruit numerous positive curvature-sensing proteins, such as F-BAR- (e.g., FBP17, FCHo2) and N-BAR- (e.g., amphiphysins, endophilins) domain proteins, epsin N-terminal homology (ENTH)-domain-containing proteins (e.g., epsins, Ap180), and Eps15 homology (EH)-domain-containing proteins (e.g., EHDs) (12). Preferential enrichment of curvature-sensing proteins leads to initiation and activation of vital cellular processes. Furthermore, the shape, size, and height of surface topography can regulate the value (i.e., diameter of curvature) and the sign (i.e., positive or negative curvature) of imprinted membrane curvature to induce different biological responses.

Herein, we briefly introduce the physicochemical concepts of membrane curvature and the curvature formed at the nano-bio interface; then we review and discuss recent advances in the nano-bio interface, focusing on the multifaceted role that surface topography plays in membrane curvature-mediated signaling pathways as they pertain to a number of biological processes, including cell adhesion, migration, differentiation, and other membrane-related events at subcellular scales.

## ENERGETICS OF MEMBRANE BENDING

Cell membranes are elastic (13). To induce curvatures on the membrane, cells have to overcome the activation energy of membrane bending. Deformation of bilayer membranes is dictated by several physical parameters, such as membrane tension, bending modulus, and the entropic contributions of lipid molecules (14–17). Couplings among individual lipid molecules and between two lipid leaflets are also implicated in membrane-curvature sorting (18, 19). The presence of membrane-associated proteins, cholesterol, and biochemical modifications to lipid molecules further adds complexity to these parameters, making cell membranes more resistant to deformation compared to simple lipid bilayer models such as unilamellar vesicles (20, 21). In cells, curvatures on the plasma membrane are primarily induced by membrane-associated proteins, despite the fact that the chemical structure and the sorting of lipid molecules, such as the size of hydrophilic head groups and hydrophobic hydrocarbon chains, also play a nontrivial role in membrane curvature generation (22, 23). In this review, we mainly focus on protein-mediated curvature sensing and induction.

## MECHANISMS OF PROTEIN-MEDIATED CURVATURE SENSING AND GENERATION

**Figure 1:**
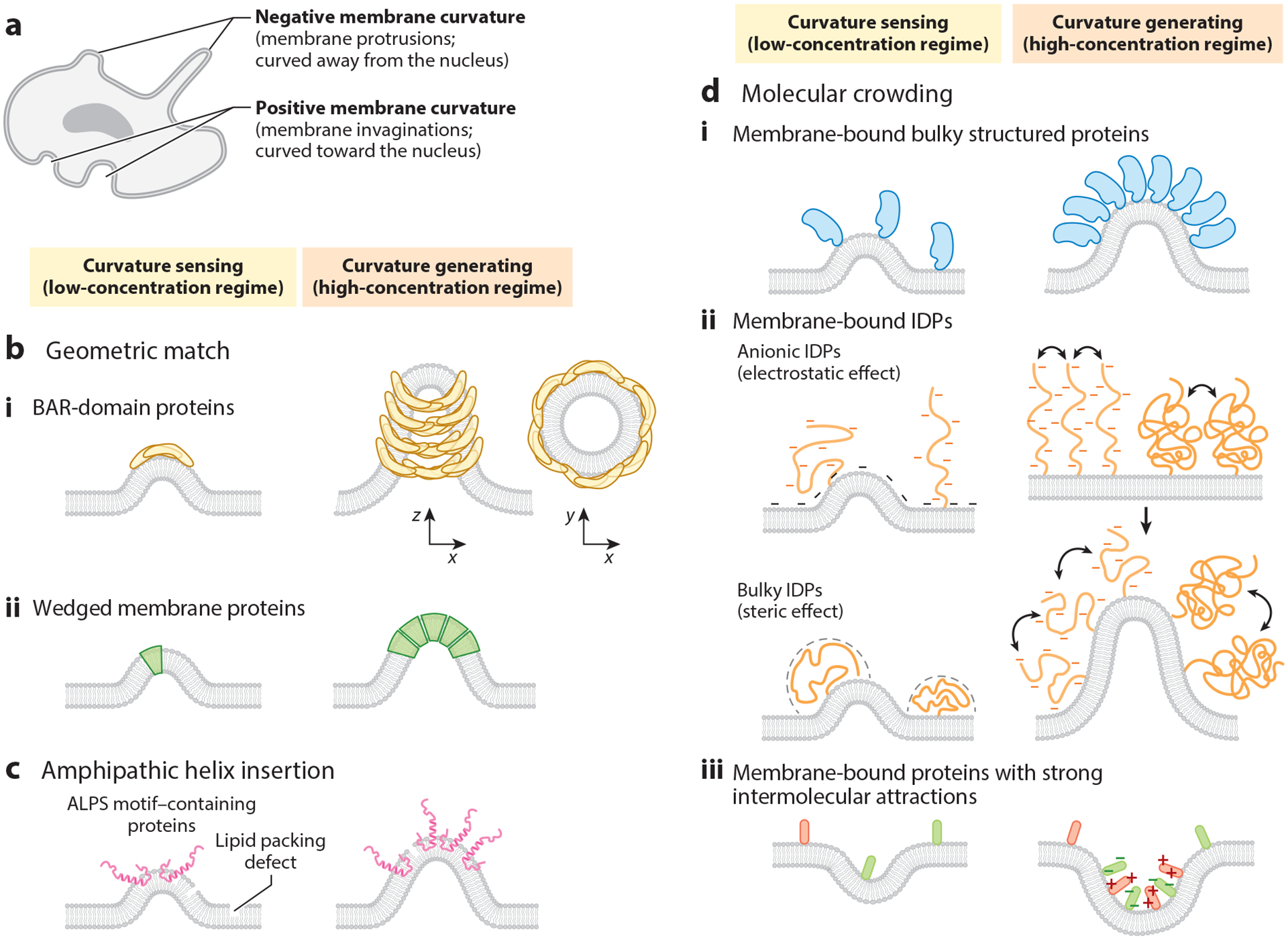
Mechanisms of protein-mediated membrane curvature sensing and generation in live cells. (*a*) Definition of positive and negative membrane curvatures in cells. (*b*) BAR-domain proteins (*i*) and wedged membrane proteins (*ii*) sense and induce membrane curvature via geometric match mechanism. (*c*) Amphipathic lipid packing sensor (ALPS) motif–containing proteins sense and induce membrane curvature by inserting their amphipathic helices into lipid-packing-defect regions. (*d*) Membrane-bound bulky structured proteins (*i*), intrinsically disordered proteins (IDPs), charged and/or bulky (*ii*), and proteins with strong intermolecular attractions (*iii*) sense and generate membrane curvature through molecular crowding mechanism. In low-concentration regimes, proteins exhibit curvature-sensing capabilities, while they are able to induce membrane curvature in high-concentration regimes. Figure adapted from images created with BioRender.com.

The mechanistic insight of membrane curvature sensing and generation has been well investigated and reviewed (12, 24–26). Cell membranes can be positively or negatively curved. Positive membrane curvature, or membrane invagination, forms when the membrane is curved toward the interior (inward) of a cell, while negative curvature, or membrane protrusion, forms when the membrane is curved away from the cellular interior (outward) (Figure 1a). Membrane curvature can be sensed and generated via diverse mechanisms, and curvature sensing and generation are tightly coupled. In general, proteins sense and preferentially bind to curved membranes at lower concentrations, while at elevated concentrations, they tend to oligomerize to bend cell membranes. Various molecular mechanisms adopted by proteins to sense and generate membrane curvature include (*a*) geometric match between curvature sensors and lipid membranes (Figure 1b; Table 1), (*b*) lipid packing defects making curvature sensor docking accessible (Figure 1c; Table 1), and (*c*) the molecular crowding of intrinsically disordered proteins (IDPs) or bulky structured proteins on cell membranes (Figure 1d; Table 1). Notably, a single type of protein may simultaneously employ multiple mechanisms to detect curved membranes and drive membrane bending. In this section, we discuss three major classes of proteins that exhibit both curvature-sensing and -generating capabilities. The functions and downstream pathways of these curvature-related proteins are discussed in later sections.

### Geometric Match Between Curvature Sensors and Lipid Membranes

The large BAR-family proteins are cytoplasmic curvature sensors that detect and bind to anionic curved membranes with matching curvature through their cationic, crescent-shaped BAR domains (see the left side of Figure 1b, subpanel *i*) (27–31). According to their intrinsic structure and preferred curvature, BAR-family proteins can be further categorized into N-BAR, F-BAR, and I-BAR subfamilies. Broadly speaking, N-BAR and F-BAR proteins preferentially sense and generate positive membrane curvature, while I-BAR proteins detect and induce negative curvature. When BAR proteins are locally concentrated on the cell membrane, they oligomerize and tubulate the membranes to initiate a number of vital signaling pathways (see the right side of Figure 1b, subpanel *i*).

Other proteins also sense and induce membrane curvature based on the geometry of plasma membranes (21, 24). Conical, wedge-shaped transmembrane proteins, including G protein-coupled receptors (GPCRs) (32, 33), KvAP, KvChim (voltage-dependent K^+^ channels) (34, 35), and Piezos (mechanosensitive ion channels) (36–40), preferentially partition into the curved segments of cell membranes with matching intrinsic curvature (see the left side of Figure 1b, subpanel *ii*). For example, GPCRs tend to accumulate in membrane protrusions with negative curvature, while KvAP, KvChim, and Piezos are preferentially enriched at positively curved membranes (36). Moreover, clustering of both KvChim (35, 41) or Piezos (41–44) has been reported to bend membranes (see the right side of Figure 1b, subpanel *ii*), although GPCRs have not been documented to demonstrate curvature-generating capabilities.

### Lipid Packing Defects Increase Accessibility for Docking of Curvature Sensors

Several cytoplasmic peripheral membrane proteins sense and generate membrane curvature by inserting their amphipathic helices (AHs) into the accessible, lipid packing defects within the inner leaflets of curved membranes (Figure 1c, *left*) (26). These include ArfGAP1, coat protein complexes (e.g., COPI), α-synuclein, ENTH-family proteins (e.g., epsins), EHD-family proteins (e.g., EHDs), and several N-BAR proteins (e.g., amphiphysins, endophilins) (45–48). More specifically, the ALPS (amphipathic lipid packing sensor) motif of several AH-containing proteins harbors polar (and/or positively charged) residues and nonpolar residues that allow the proteins to bind the hydrophilic head groups and the aliphatic hydrocarbon chains of lipids, respectively. At high local densities, insertion of AHs also induces membrane deformation via clustering within the inner leaflets of membrane lipid bilayers, which triggers membrane trafficking and several other downstream signals (Figure 1c, *right*).

### Molecular Crowding of Intrinsically Disordered Proteins or Globular Proteins on Cell Membranes

Membrane-bound polymer-like IDPs or bulky structured proteins are also capable of sensing and inducing membrane curvature (Figure 1d). The curvature-sensing and -generating capabilities of the proteins in this class are primarily governed by the intermolecular steric repulsions (namely, configurational/conformational entropy) on the bilayer membrane. Membrane-bound IDPs such as MUC1, epsins, and AP180 (49–52) and bulky structured proteins (53) all preferentially partition into curved membranes with convex curvature (curved toward the proteins), through which they reduce intermolecular repulsions and increase configurational entropy (steric effect) (see the left side of Figure 1d, subpanels *i*,*ii*). When the membrane-bound protein tethers are charged (mostly anionically), electrostatic interactions among the proteins and between proteins and anionic lipids also determine the curvature preference (electrostatic effect) (54). For instance, preferential accumulations of negatively charged IDPs at convex membranes reduce Coulombic repulsions as well as free energy among IDPs and the anionic membrane. At high surface density, the steric pressure allows protein tethers to bend the membrane toward them (convex curvature), leading to an increase in entropy and decrease in free energy (see the right side of Figure 1d, subpanels *i*,*ii*). However, depending on whether intermolecular attractions (electrostatic attraction, dipole-dipole interaction, cation-π interaction, etc.) or repulsions dominate, protein tethers might curve the membrane toward them (convex) or away from them (concave) (see Figure 1d, subpanel *iii*). For instance, FUS [a heterogeneous nuclear ribonucleoprotein (hnRNP)] and hnRNPA2, which possess intrinsically disordered RGG/RG domains and tend to undergo liquid-liquid phase separation (LLPS), preferentially sense concave curvature (54, 55). Upon oligomerization at high concentrations, FUS condensates can push bilayer membranes outward, resulting in concave curvatures (54, 55). Noticeably, according to the location (extracellular or intracellular) and orientation of membrane-bound proteins, the convex or concave curvature they sense and induce can be either a positive or a negative membrane curvature (Figure 1a). It has also been shown that synergy between steric (entropic) and electrostatic mechanisms of IDPs (51), or between intrinsically disordered regions (IDRs) and structured domains of proteins (52), enables them to sense and induce membrane curvature in a more elaborate manner.

## PHYSICAL CHEMISTRY AT THE NANO-BIO INTERFACE

Cells interact with nanomaterials or the nanotopographical features on substrates mainly through noncovalent interactions, such as ionic interactions, hydrogen bonding, dipole-dipole interactions, hydrophobic interactions, etc. (56). The physical properties (e.g., the geometry, dimension, and spatial arrangement of nanotopographical features, as well as substrate stiffness, etc.) and chemical properties (e.g., hydrophilicity/hydrophobicity, chemical functional groups, coatings on the surface of nanostructures, etc.) of nanotextures significantly modulate cell adhesions on nanopatterned substrates (6, 57).

When cells adhere to nanostructured substrates, the surface nanotopography imprints local nanoscale membrane curvatures on cell membranes. Experimentally, focused ion beam scanning electron microscopy (FIB-SEM)-based studies have revealed that cell membranes tightly wrap around vertical nanostructures protruding from substrates (e.g., nanoneedles, nanopillars, nanowires) (58–60). A very recent numerical simulation work by Jin and colleagues (61) describes the evolution of membrane curvature during cell docking onto vertical nanopillar arrays. The authors prove that ligand-receptor binding energy at the cell-nano interface can surmount the energy barrier of membrane bending. In agreement with previous experimental observations (62–64), the authors also identify that surface topography and membrane tension both regulate cell adhesions on vertical nanostructures, with smaller nanopillar diameters, sparser nanopillar density, and lower membrane tensions supporting faster cell attachment and membrane deformation.

## NANOSTRUCTURED PLATFORMS FOR STUDYING MEMBRANE CURVATURE AND CURVATURE-SENSING PROTEINS

**Figure 2:**
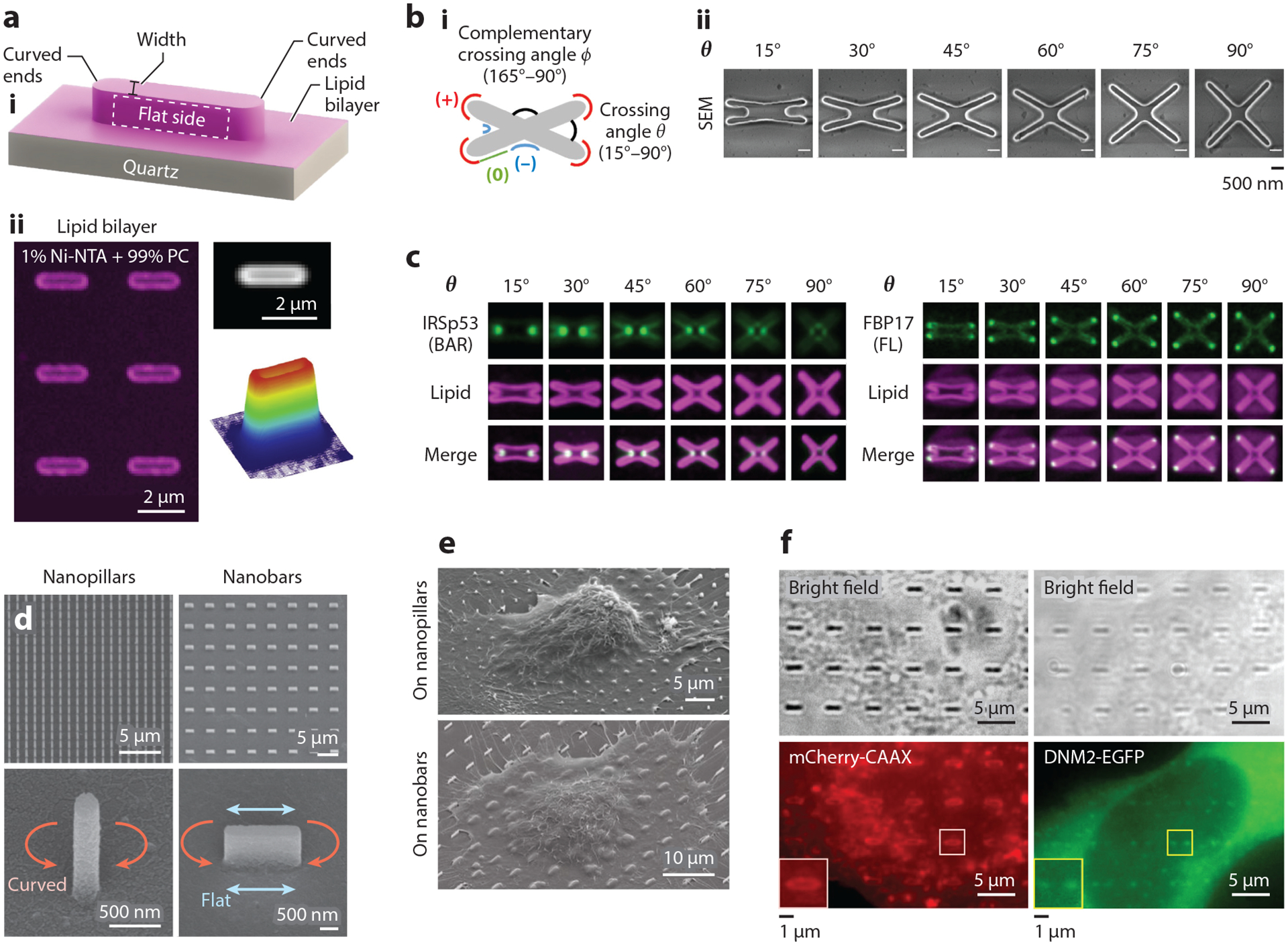
Nanostructured platforms for studying membrane curvature and curvature-sensing proteins in vitro and in live cells. (*a*, *i*) Schematic illustration of a nanobar coated with supported lipid bilayer (SLB). (*ii*) Fluorescence image, averaged fluorescence image, and 3D surface plot of SLB on nanobar arrays. Panel *a* adapted from Reference 69. (*b*) Schematic illustration of nanoX (*i*) and scanning electron microscopy (SEM) images of individual nanoX (*ii*) with varying inner angles. Panel *b* adapted from Reference 66. (*c*) Averaged fluorescence images of GFP-tagged IRSp53 BAR domain [IRSp53 (BAR)-GFP] and full-length GFP-labeled FBP17 [FBP17 (FL)-GFP] fluorescence signals on SLB-coated gradient nanoX arrays. The lipid bilayer is visualized via Texas Red labeling. IRSp53 (BAR) displays a strong preference for negative membrane curvature induced at the inner grooves of nanoX, while FBP17 preferentially accumulates at the arm ends of nanoX where positive curvature is imprinted. Panel *c* adapted from Reference 66. (*d*) SEM images of quartz-based nanopillar (*left column*) and nanobar (*right column*) arrays. Panel *d* adapted from Reference 72. (*e*) SEM images of U2OS cells cultured on nanopillar (*top*) or nanobar (*bottom*) arrays, illustrating deformation of plasma membranes by these vertical nanostructures. Panel *e* adapted from Reference 112. (*f*) Bright-field (*top row*) and live-cell fluorescence images (*bottom row*) of U2OS cells expressing a plasma membrane marker (mCherry-CAAX) (*bottom left*) or EGFP-tagged dynamin-2 (DNM2-EGFP) (*bottom right*) cultured on nanobar arrays. mCherry-CAAX wraps around nanobars without exhibiting curvature preference, while DNM2-EGFP displays a clear preference for nanobar ends where positive curvature is induced. Panel *f* adapted from Reference 72.

Various biophysical methods have been developed to study membrane curvature at the nano-bio interface. In particular, nanofabrication offers precise engineering of surface topography of substrates that impart well-controlled membrane curvature on both supported lipid bilayers (SLBs) and mammalian cells (49, 65–68). In vitro studies using SLBs generated on nanofabricated substrates consist of incubating the SLB-coated, nanosubstrate platform with either cell lysate containing curvature-sensing proteins or purified curvature-sensing proteins (66–68). Hsieh and colleagues (67) generated SLBs on fabricated wavy substrates with alternating regions of positive and negative curvature and incubated them with purified ENTH, N-BAR domains of BIN1 or endophilin, or cholera toxin B subunit (CTxB) to probe their curvature-sensing properties. Furthermore, a distinct advantage of nanofabrication lies in the engineering of well-defined membrane curvature. Nanofabrication generates unique topographical shapes to study positive and negative curvature and the effect of varying curvature diameters. One study by Su and colleagues (68) utilized nanobar structures with SLBs (Figure 2a) and determined that the curvature-sensing ability of FBP17 derives from the IDR rather than the F-BAR domain alone. Another study by Lu and colleagues (66) developed a nanostructure-based, curvature-sensing platform, termed NanoCurvS, consisting of X- or U-shaped nanostructure arrays (nanoX and nanoU, respectively) to induce curvature on SLBs formed atop the substrates. The nanoX structures allowed for the study of the positive curvature generated by the curved ends of the arms and the negative curvature generated by the inner crossings of the X-shape, while the straight arms of the X served as an internal control (Figure 2b). Varying the inner angles of the nanoX structures, which created a range of curvature diameters, subsequently allowed for investigations into the negative- and positive-curvature-sensing properties of BAR-domain proteins, such as IRSp53 and FBP17, respectively (Figure 2c). Also worth noting is nanoparticle-induced membrane curvature on SLBs, a method that circumvents more complex nanofabrication demands (70, 71).

Complementary to in vitro methods, membrane curvature studies in mammalian cells better capture the complexity of living systems. Culturing mammalian cells on nanofabricated substrates, including nanopillars, nanobars, and nanoridges, allows a precise and local manipulation of the plasma membrane (Figure 2d,e) (72). Nanofabricated substrates are biologically compatible with live cells, making them useful for characterizing the dynamics of curvature-sensing proteins. For example, Galic and colleagues (73) show that membrane curvature induced by cone-shaped nanostructures results in the recruitment of N-BAR domains to live-cell membranes. Additionally, we note that these nanostructures are optically compatible with numerous microscopy techniques. Fluorescence microscopy of cells plated on nanofabricated substrates has allowed for both live-and fixed-cell studies. Through immunostaining and/or overexpression, proteins of interest in cells are easily visualized to examine their curvature preferences (Figure 2f) (72, 74, 75), while techniques such as expansion microscopy (76) and super-resolution microscopy (77) have also been employed to visualize the localization of curvature-related proteins within fixed cells on nanostructured substrates. Finally, to circumvent the static nature of fabricated nanostructures, materials whose shape can be modified by light have also been developed to dynamically reshape membrane curvature (78). In the last decade, many studies have utilized such engineered substrates to study membrane curvature and curvature-induced intracellular signaling, some of which have led to surprising new discoveries. In the following sections, we review recent advances utilizing nanofabricated substrates to investigate a diverse range of intracellular signaling and cellular processes occurring at the nano-bio interface.

## PLASMA MEMBRANE CURVATURE REGULATES CELL ADHESIONS AT THE NANO-BIO INTERFACE

### Surface Nanotopography Modulates Cell Adhesions

As an indispensable process for cells to anchor themselves to substrates, cell adhesion has been extensively studied and implicated in many other vital cellular physiological processes. Nanoscale surface topography has a significant impact on cell adhesions (5, 79–81). Thanks to advances in surface nanopatterning, substrates with diverse surface nanotopographical features can be precisely engineered (82, 83). Interestingly, surface nanotopography has been documented to both promote and impede cell adhesions, depending on the aforementioned physicochemical properties of nanostructured substrates and cell type (84–86). For example, using nanogroove-patterned substrates, several studies show that focal adhesions and actin stress fibers are enhanced and orient parallel to the nanoridged patterns (87–89). On the other hand, using nanopillars (62, 63, 90, 91), nanocolumns (92), or nanopores (93), studies have identified that cells spread less and form fewer focal adhesions and stress fibers on these nanotopographical features, suggesting an inhibitory effect on cell adhesions (63, 87–92, 94). To delve into the mechanism, Li and colleagues (63) demonstrate that nanoscale surface topography attenuates cell adhesions by stimulating endocytosis of integrin β1 in human mesenchymal stem cells and U2OS cells. In addition, the authors note that cellular response to nanostructured rigid quartz substrates is similar to the response to soft substrates, as cell stiffness and membrane tension are reduced on nanopillar arrays to a similar extent as on soft hydrogels. Furthermore, Beckwith and colleagues (62) show that at the same nanopillar diameter (~100 nm), NIH-3T3 cells (mouse embryonic fibroblasts) preferentially stay on top of nanopillars when the spacing is dense (≤1 μm) but tightly wrap around nanopillars and adhere strongly to the substrate bottom when the nanopillars are sparsely distributed (≥2 μm). A very similar phenomenon has also been observed in U2OS cells (90) and rat embryonic cortical neurons (64).

### Membrane Curvature Mediates the Formation of Curved Adhesions at the Nano-Bio Interface

Integrin-mediated focal adhesions form abundantly on tissue culture plates and have been extensively characterized (95–97). The formation of focal adhesions requires rigid extracellular environments. Consequently, focal adhesions do not form or are sparse on soft hydrogels and soft 3D ECM. Vogel & Sheetz (98) proposed in 2006 that the plasma membrane curvature induced by membrane wrapping around ECM fibers could play a role in 3D cell adhesion. This hypothesis has been recently tested and corroborated by our and other groups.

**Figure 3:**
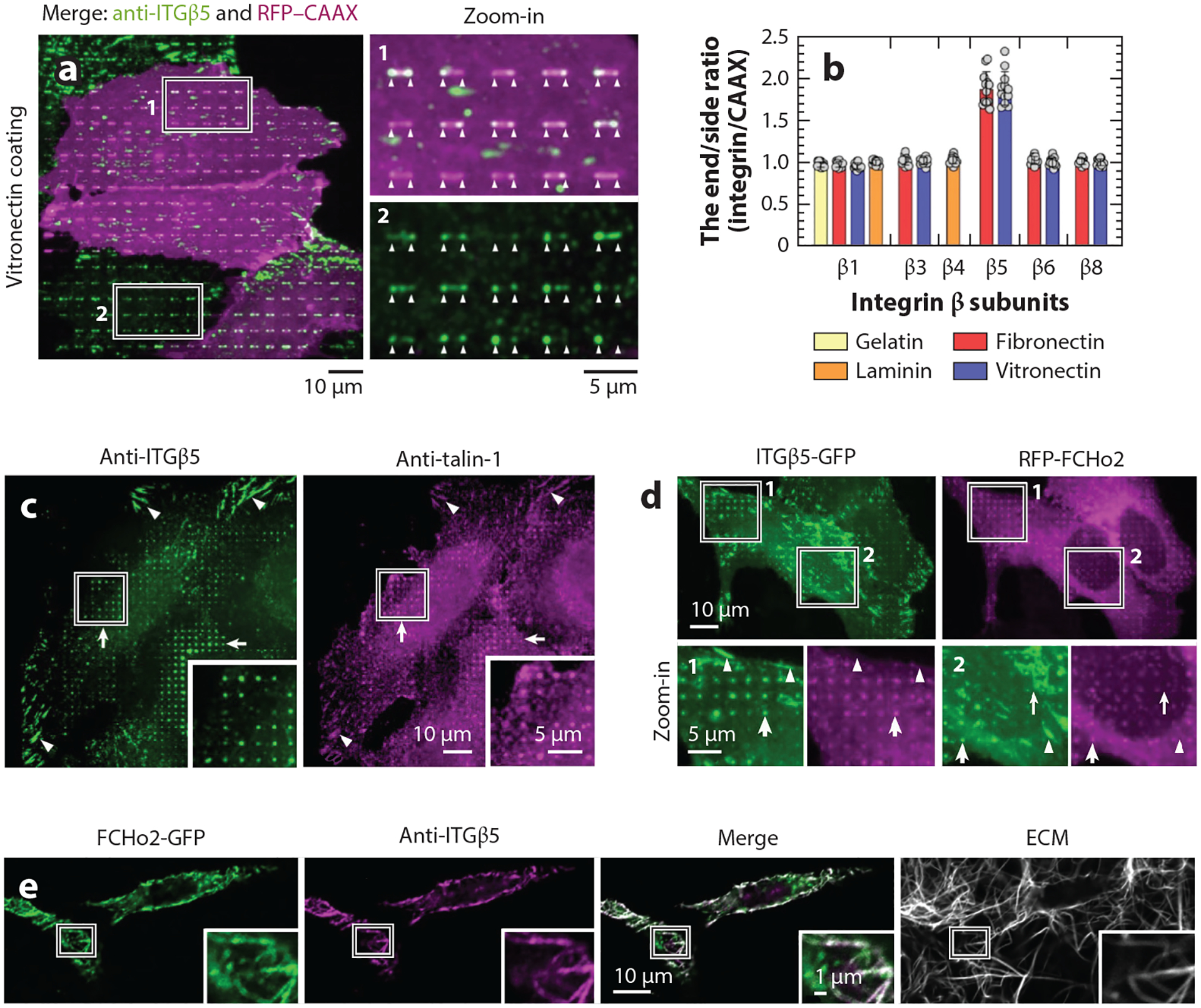
Plasma membrane curvature regulates cell adhesions at the nano-bio interface. (*a*) Fluorescence images of U2OS cells cultured on vitronectin-coated nanobar arrays. The plasma membrane marker RFP-CAAX wraps uniformly around nanobars, while integrin β5 (ITGβ5) shows clear preference for nanobar ends. (*b*) Quantification of the curvature preferences of various integrin β isoforms on nanobar substrates coated with their corresponding extracellular ligands. Curvature preference is acquired by measuring the nanobar end/side intensity ratios, normalized to the end/side ratios of the membrane marker RFP-CAAX. Only ITGβ5 exhibits a preference for positive membrane curvature. (*c*) Anti-ITGβ5 spatially correlates with anti-talin-1 in curved adhesions formed at vitronectin-coated nanopillars (*white arrows*) and in focal adhesions on flat areas (*white arrowheads*). (*d*) GFP-labeled ITGβ5 (ITGβ5-GFP) positively correlates with RFP-FCHo2 at vitronectin-coated nanopillar locations (*white arrows*) but not in focal adhesion patches (*white arrowheads*). (*e*) Curved adhesions (marked by both ITGβ5 and FCHo2) are abundant in 3D vitronectin-enriched, soft fibrous environments. Abbreviation: ECM, extracellular matrix. Figure adapted from Reference 99 (CC BY 4.0).

Using nanopillars and nanobars, Zhang and colleagues (99) recently discovered a new class of integrin-based cell adhesions, termed curved adhesions, which exclusively form at curved plasma membranes. The authors characterize that among many β integrin isoforms, only integrin β5 preferentially accumulates at curved membranes induced either by vertical nanopillars or by nanobars (Figure 3a,b). The formation of curved adhesions requires the binding of extracellular ligands such as vitronectin or fibronectin. Using gradient nanobar arrays that imprint a broad range of membrane curvature diameters (100 nm–5 μm) on cells, it is revealed that integrin β5 is sensitive to positive membrane curvatures up to ~3 μm in diameter, coinciding with the diameters of ECM fibers in physiological environments. Curved adhesions recruit the mechanophore talin and bear mechanical forces, although the forces are much lower than those of focal adhesions (Figure 3c) (99, 100). In terms of molecular composition, curved adhesions share a subset of constituents with focal adhesions, including talin, paxillin, and zyxin, but do not contain vinculin or pFAK, which are crucial components of focal adhesions. A curvature-sensing protein FCHo2, which had long been described as an endocytic protein, is uniquely present in curved adhesions. FCHo2 interacts with and recruits integrin β5 to curved membranes (Figure 3d). Functionally, curved adhesions form abundantly on soft 3D ECM fibers and enable cancer cell migration in a 3D, vitronectin-enriched collagen fibrous network (Figure 3e,d). Silencing the key components integrin β5 and/or FCHo2 drastically compromises cell migration in a 3D ECM.

### Membrane Curvatures Serve as Hot Spots for Endocytosis

As an essential cellular pathway for internalizing extracellular ligands and cell surface receptors, endocytosis has been well investigated in recent decades. Various classes of cellular uptake mechanisms have been identified, including clathrin-mediated endocytosis (CME) (101), caveolin-mediated endocytosis (102), fast endophilin-mediated endocytosis (FEME) (103), macropinocytosis (104), and phagocytosis (105). To initiate endocytosis, membrane bending (i.e., curvature generation) is the committed, rate-limiting step subject to tight regulation (25). Recent studies show that endocytosis preferentially occurs at curved plasma membrane locations.

Through mathematical modeling, Gao and colleagues (106) elucidate that receptor-ligand binding causes local membrane deformation by wrapping around ligand-coated nanoparticles. The elevated elastic energy of membranes and the energy associated with reduced configurational entropy are compensated by ligand-receptor binding energy. Experimentally, Peetla and colleagues (107) further demonstrate that biomechanical and thermodynamic interactions between nanoparticles and plasma membranes assist the uptake of nanoparticles into cancer cells. Stronger nanoparticle-membrane interactions trigger cellular internalization by surmounting the energy barrier of membrane deformation. Several independent studies state that induced membrane curvature by vertical nanostructures can promote endocytosis by lowering the energy barrier of membrane bending. High-aspect-ratio nanostructures, such as nanoneedles, nanopillars, nanowires, and nanostraws, increase the delivery of biomolecules (proteins, nucleic acids, etc.), drugs, and nanoparticles into cells or live animals (83, 108). Chiappini and colleagues (109) have established mesoporous silicon nanoneedles for efficient delivery of cell-impermeable quantum dots into mice. From the same group, Gopal and colleagues (59) further demonstrate the use of porous silicon nanoneedles to deliver cargo through different endocytosis pathways, including transferrin for CME, CTxB for caveolar endocytosis, and 10–70-kDa dextran for macropinocytosis.

**Figure 4:**
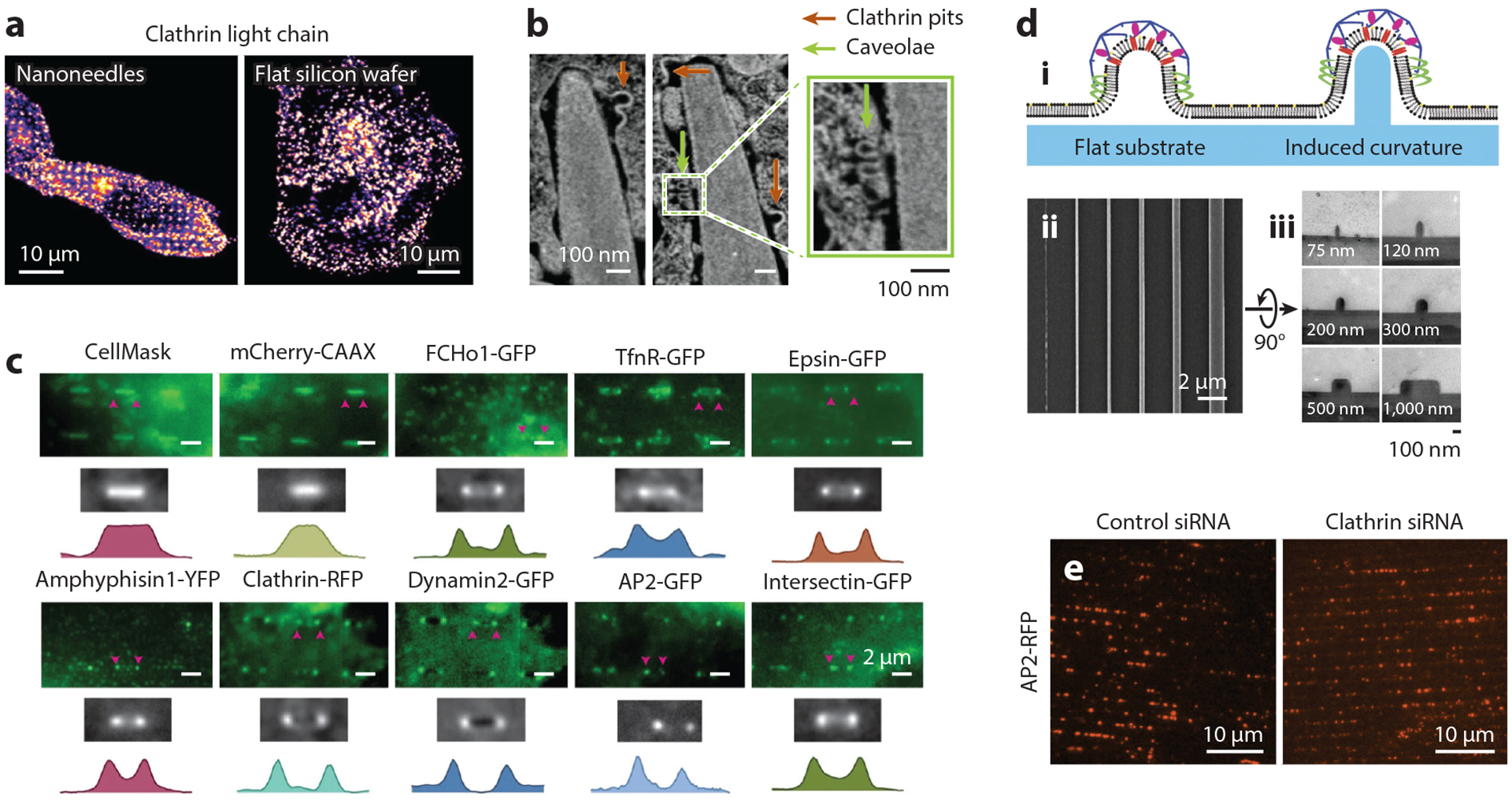
Membrane curvatures serve as hot spots for endocytosis. (*a*) Confocal images of the basal membrane of human mesenchymal stem cells cultured on nanoneedle arrays (*left*) or flat silicon wafers (FSW) (*right*). Clathrin strongly accumulates at nanoneedles, yet it is randomly distributed when cells are plated on FSW. (*b*) Focused ion beam scanning electron microscopy images showing accumulations of clathrin-coated pits and caveolae around nanoneedles. Panels *a* and *b* adapted from Reference 59 (CC BY 4.0). (*c*) Single fluorescence image (*top*), averaged nanobar fluorescence image over hundreds of nanobars (*middle*), and intensity profiles (*bottom*) of various endocytic proteins expressed in SK-MEL-2 cultured on nanobar arrays. Panel *c* adapted from Reference 74. (*d*, *i*) Schematic illustration of clathrin-coated pit formation on flat or nanoridge surfaces. Scanning electron microscopy (SEM) (*ii*) and transmission electron microscopy (TEM) (*iii*) images of a nanoridge substrate. (*e*) AP2 preferentially accumulates at membrane invaginations induced by nanoridges in control (*left*) or clathrin-depleted (*right*) MDA-MB-231 cells. Panels *d* and *e* adapted with permission from Reference 75. Abbreviation: siRNA, small interfering RNA.

At the molecular level, nanoneedle-induced membrane invaginations locally enrich clathrin-coated pits and caveolae to activate clathrin- and caveolin-dependent endocytosis, respectively (Figure 4a,b). In contrast, both proteins are randomly distributed in the cytoplasm when cells are cultured on flat silicon wafers (FSW) (Figure 4a,b). Zhao and colleagues (74) reveal that nanoscale membrane curvature induced by quartz-based vertical nanopillars and nanobars can efficiently promote CME by recruiting several endocytic proteins, including clathrin, dynamin, AP2, amphiphysin-1, FCHo1, and epsin-1, to positively curved membranes of SK-MEL-2 cells (Figure 4c). Moreover, curved membranes serve as hot spots for the initiation and turnover of endocytic events, allowing more efficient uptake of targets. A follow-up work by Cail and colleagues (75) further unveils that with the aid of membrane curvature induced by vertical nanoridges (Figure 4d), CME in MDA-MB-231 cells can be achieved even with the depletion of clathrin. Induced membrane invaginations recruit AP2 and dynamin and rescue CME dynamics in the absence of clathrin (Figure 4e). Moreover, consistent with the observation by Zhao and colleagues, Cail and colleagues (75) also identified that sharp positive membrane curvature (<300-nm diameter of curvature) induces the preferential accumulation of several CME proteins, including clathrin, AP2, epsin-1, and dynamin.

### Membrane Curvature Induces Local Actin Polymerization and Reorganization

Nanoscale surface topography regulates local actin reorganization and polymerization by modulating membrane curvature (110, 111). Unlike long, linear actin stress fibers, which are mainly mediated by formins, the population of F-actin enriched at the membrane curvature is mostly branched actin filaments mediated by the nucleator Arp2/3 (78, 112–114). Several works highlight the roles of phospholipids, membrane curvature, and curvature-sensing BAR-domain proteins in actin polymerization (114–116). Through in vitro, liposome-based assays, these studies demonstrate that phosphoserine (PS) and phosphatidylinositol 4,5-bisphosphate [PI (4, 5)P_2_] in lipid membranes synergize with membrane curvature to promote actin polymerization in a manner dependent on Arp2/3, CDC42 (a Rho-family small GTPase), and N-WASP (an Arp2/3 nucleation promoting factor). The work by Takano and colleagues (114) shows that TOCA-subfamily F-BAR proteins, including FBP17, CIP4, and TOCA-1, serve as molecular bridges to engage actin nucleators and their effectors at curved membranes. Moreover, the N-BAR-domain protein SNX9 has also been implicated in curvature-regulated actin polymerization (115, 116).

**Figure 5:**
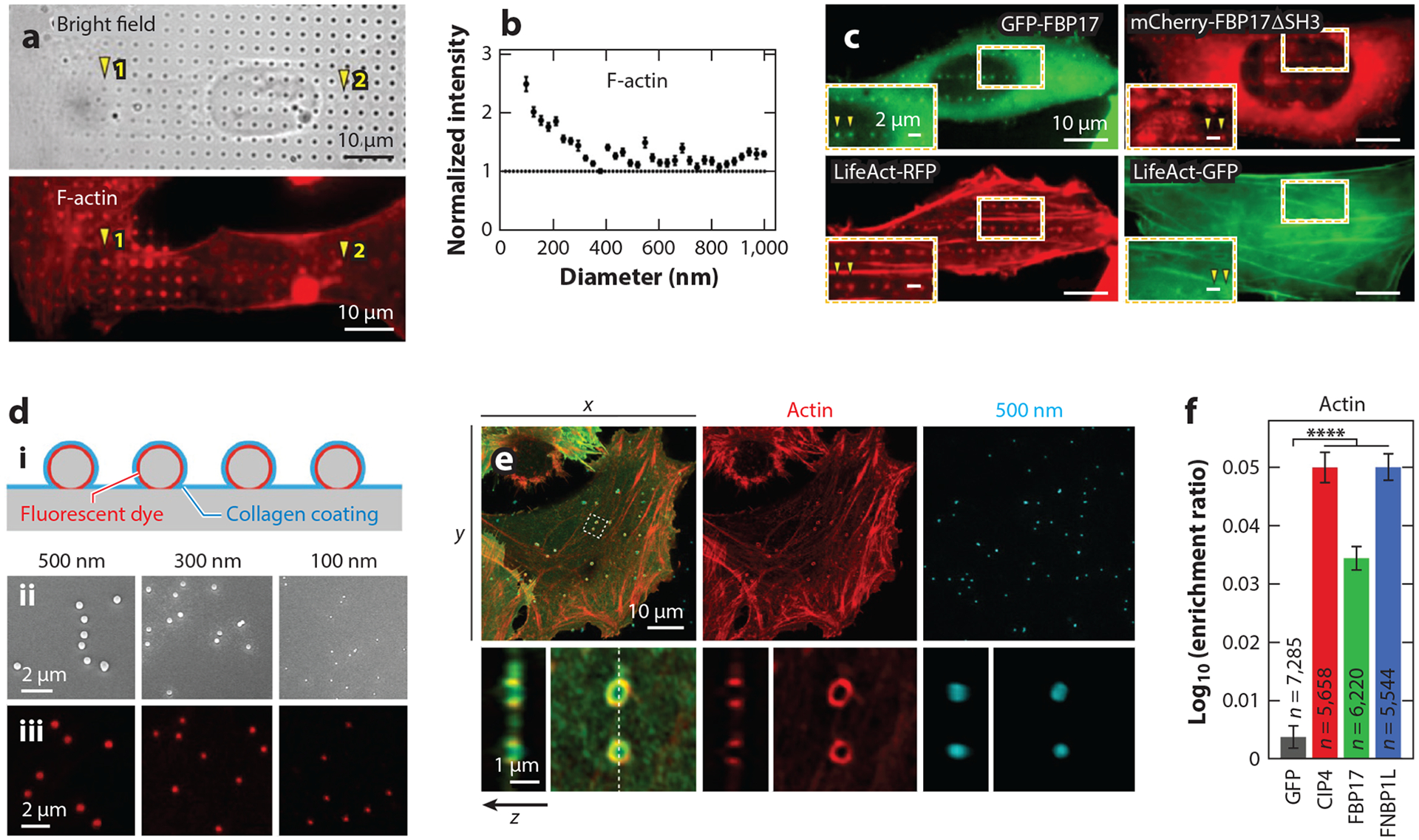
Membrane curvature induces local actin polymerization and reorganization. (*a*) Bright-field (*top*) and fluorescence image (phalloidin staining) of F-actin (*bottom*) in U2OS cells cultured on gradient nanopillar arrays. F-actin strongly accumulates at smaller nanopillars that induce sharper positive membrane curvature. (*b*) Quantification of the curvature preferences of F-actin on gradient nanopillar arrays. F-actin responds to sharp positive curvature induced by smaller nanopillars (<400 nm in diameter). (*c*) Co-transfection of LifeAct (an F-actin probe) with either full-length FBP17 or FBP17 (ΔSH3) in U2OS cells cultured on nanobar arrays. Both F-actin and full-length FBP17 preferentially accumulate at nanobar ends. However, F-actin exhibits a much reduced preference for nanobar ends when cells are coexpressed with FBP17 (ΔSH3). Panels *a–c* adapted from Reference 112. (*d*, *i*) Schematic illustration of nanostructured substrates using silica colloids of different diameters. (*ii*) Scanning electron microscopy and (*iii*) confocal images of silica colloids adsorbed on coverslips. Substrates are labeled with ATTO 390 for visualization. (*e*) Confocal images of HeLa cells cultured on 500-nm nanostructured substrates illustrating the accumulation of F-actin around silica colloids. Green signals represent the membrane marker. (*f*) Quantification of F-actin enrichment in HeLa cells seeded on 100-nm nanostructured substrates. Coexpression of each GFP-tagged TOCA-family F-BAR protein facilitates F-actin enrichment at positively curved membranes. Panels *d–f* adapted with permission from Reference 117.

Curvature-dependent actin polymerization has been further confirmed using cell-based assays along with nanotopography-induced membrane curvature. Using quartz nanopillar and nanobar arrays, Lou and colleagues (112) demonstrate that sharp membrane invaginations (<400-nm diameter of curvature) facilitate branched actin filament polymerization (Figure 5a,b). This observation is supported by the preferential accumulations of Arp2/3, N-WASP, and cortactin (another Arp2/3 nucleation promoting factor) at a positive membrane curvature. In contrast, formin-family proteins (mDia1 and mDia2) show no curvature preference. Moreover, preferential accumulation of F-actin at membrane invaginations is largely compromised when cells are coexpressed with the dominant-negative FBP17 variant (FBP17ΔSH3) lacking the SH3 domain, the region required for N-WASP binding and Arp2/3 recruitment (Figure 5c). Using silica colloid-decorated nanostructured surfaces, a recent work by Ledoux and colleagues (117) further delves into the mechanistic details of how membrane curvature manipulates actin polymerization (Figure 5d,e). The authors screened the curvature sensitivity and sensing range of BAR-domain proteins (including N-BAR, F-BAR, and I-BAR proteins) and determined that curvature-dependent actin polymerization is primarily mediated by TOCA-subfamily F-BAR proteins, including CIP4, previously identified TOCA-1 (also known as FNBP1L), and FBP17 (Figure 5f) (114, 115).

### Membrane Curvature Modulates the Spatial Distribution of Glycocalyx

The outer surface of mammalian cells is covered with a carbohydrate-enriched coating, termed glycocalyx, which is a layer of heavily and heterogeneously glycosylated components, including glycoproteins, proteoglycans, glycolipids, and newly discovered glycoRNAs (118, 119). Due to its biophysicochemical properties, the glycocalyx can protect cells from pathogenic attack, lubricate cells, and modulate ligand binding to cell-surface receptors (118, 120–122). In numerous types of cancer cells, glycocalyx components are overexpressed with altered glycosylation levels and glycan compositions, suggesting the critical impact of the glycocalyx on cancer progression (123).

**Figure 6:**
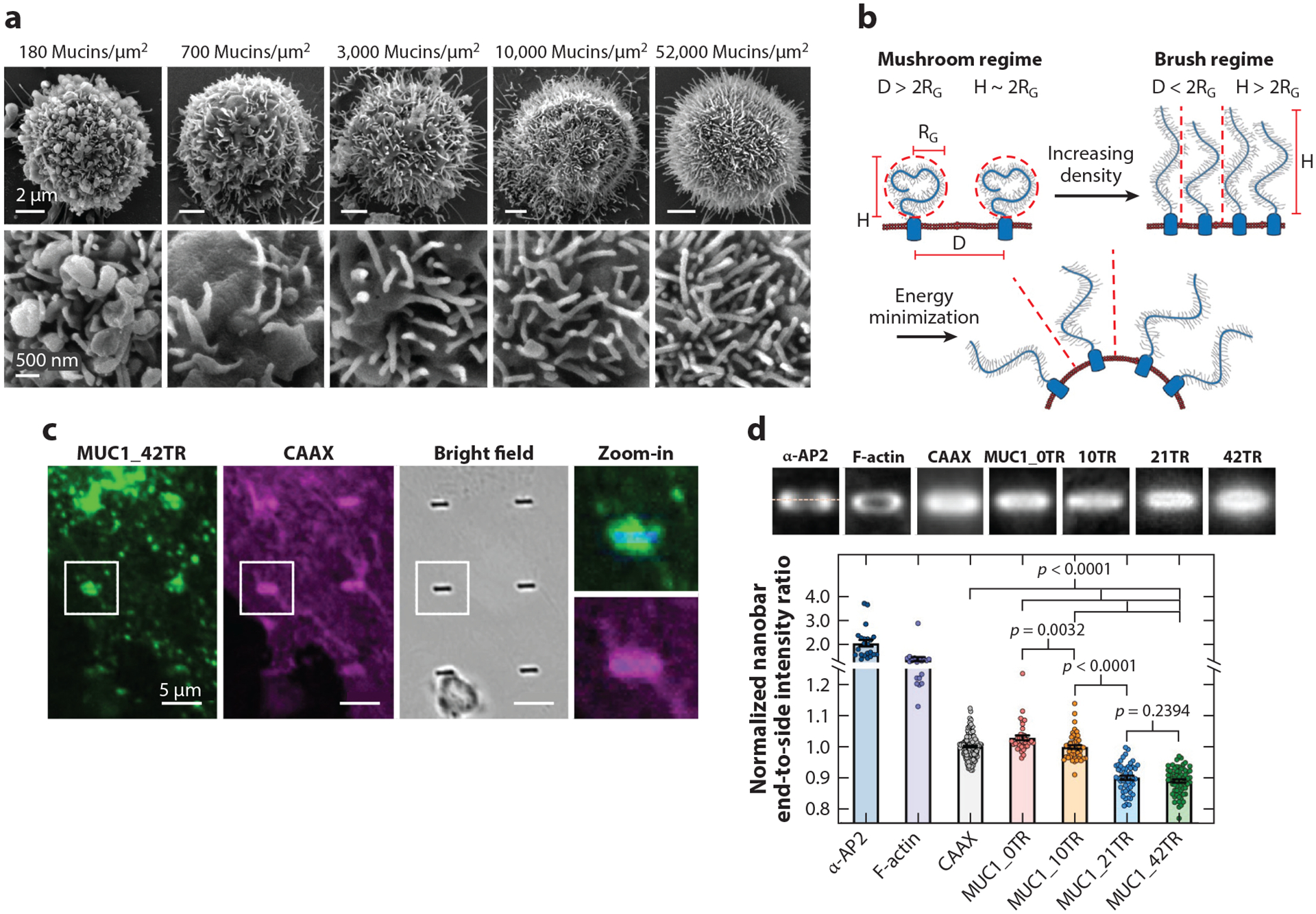
Membrane curvature modulates the spatial distribution of glycocalyx. (*a*) Scanning electron microscopy images showing the change in plasma membrane morphology from blebs to narrow tubules with increasing density of cell-surface mucins. (*b*) Polymer brush model depicting spontaneous membrane bending via molecular crowding of cell-surface glycopolymers. D is the average distance between adjacent polymers, R_G_ is the radius of gyration of polymers, and H is the height of polymers. Panels *a* and *b* adapted with permission from Reference 125. (*c*) When U2OS cells are cultured on nanobar arrays, MUC1 with the full-length ectodomain [42 tandem repeats (TR)] avoids positively curved membranes induced at nanobar ends. (*d*) Averaged nanobar fluorescence images (*top*) and quantification of the curvature preferences of AP2, F-actin, CAAX, and MUC1 with varying numbers of TR on nanobar arrays (*bottom*). Panels *c* and *d* adapted from Reference 49 (CC BY 4.0).

The glycocalyx has recently emerged as a new star in membrane biophysics due to its novel role in sculpting membrane shapes. Glycocalyx biopolymers have been documented to bend plasma membranes through generation of entropic pressure (124). An elegant study by Shurer and colleagues (125) shows that cell-surface glycopolymers such as MUC1 induce membrane protrusions (curved away from cytoplasm, namely, negative curvature). The geometry of membrane protrusions strongly depends on the surface density of glycopolymers. More specifically, at a low surface density (~180 mucins/μm^2^), glycopolymers reside in the “mushroom” regime and tend to form blebs on membranes (Figure 6a). At elevated surface densities at which glycopolymers accommodate the “brush” regime (~700–52,000 mucins/μm^2^), glycopolymers tubulate the membrane, thereby releasing steric and electrostatic repulsions among neighboring glycopolymers (Figure 6b). Combining cellular experiments with computational models, Paszek and colleagues (126, 127) argue that cancer-associated MUC1 and long synthetic glycoprotein mimetics can promote cancer cell growth and survival by facilitating integrin-mediated cell adhesions. Mechanistically, bulky mucin glycopolymers trigger integrin clustering by funneling active integrins into adhesion architectures at membrane protrusions.

Conversely, membrane curvature has also been reported to govern the spatial distribution of glycocalyx biopolymers on the membrane. By using nanobar and nanoX arrays and membrane-sculpting proteins, Lu and colleagues (49) discovered that bulky glycoproteins like MUC1 avoid positively curved membranes (i.e., invaginations) and prefer negatively curved ones (i.e., protrusions) in cells and in vitro. The avoidance of positive membrane curvature is dependent on the size and glycosylation state of MUC1 (Figure 6c). MUC1 with longer ectodomains and higher glycosylation levels displays a stronger avoidance of membrane invaginations (Figure 6d). Such avoidance of positive membrane curvature contributes to the delayed removal of MUC1 by endocytosis, resulting in a long lifetime on cell surfaces. A mechanistic study by Gollapudi and colleagues (128) further confirms that reduced internalization of MUC1 via clathrin-mediated endocytosis is attributed to the bulkier ectodomain. In this study, long and heavily glycosylated forms of MUC1 result in weaker partitioning into clathrin-coated structures where positive membrane curvature prevails. In conclusion, the glycocalyx can act as both a curvature sensor and a curvature generator.

### Membrane Curvature Regulates Mechanosensitive Ion Channels

Mechanosensitive ion channels are ionotropic transmembrane proteins that facilitate cellular response to mechanical stimuli and are involved in a number of crucial biological processes, such as touch, hearing, pain, and blood pressure regulation. Mechanical perturbations influence mechanosensitive ion channel conformation, affecting downstream biological processes. Given that the cell membrane is a geometrically dynamic bilayer subject to mechanical forces that generate local membrane curvature, numerous studies have linked mechanosensing ion channels to membrane curvature.

**Figure 7:**
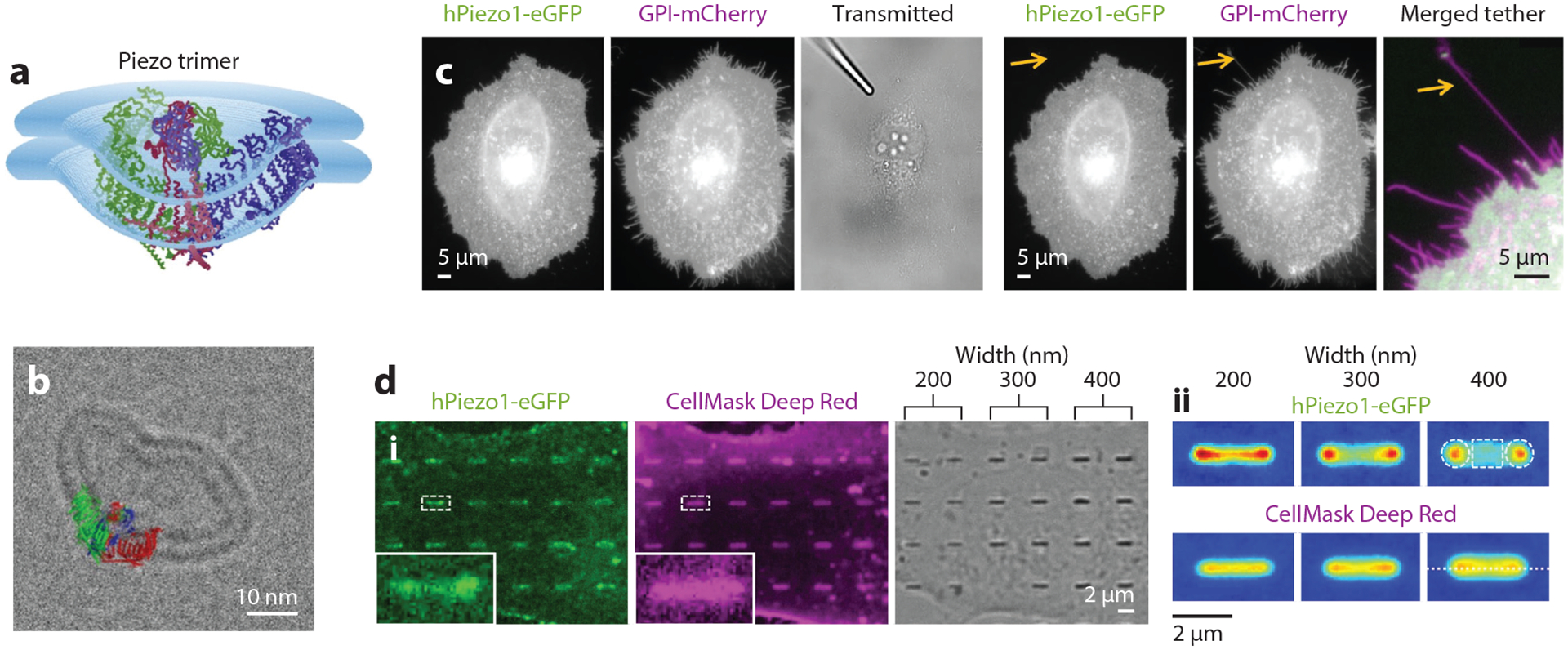
Membrane curvature regulates mechanosensitive ion channels. (*a*) Representative Cα trace model of a Piezo trimer embedded in a hemisphere-shaped membrane. (*b*) A Piezo trimer embedded in a small unilamellar vesicle with the molecular model. Panels *a* and *b* adapted from Reference 37 (CC BY 4.0). (*c*) Fluorescence images of a HeLa cell coexpressing human (h) Piezo1-eGFP and GPI-mCherry (glycosylphosphatidylinositol). Upon cell membrane pulling by a micropipette (see *orange arrows* in the *right subpanels*), Piezo1 is excluded from the membrane protrusion with sharp negative curvature. (*d*) Fluorescence images (*i*) and heatmaps of averaged nanobar fluorescence images (*ii*) of U2OS cells expressing hPiezo1-eGFP cultured on nanobar arrays. Cell membranes are visualized by CellMask staining. Panels *c* and *d* adapted from Reference 36 (CC BY 4.0).

Coste and colleagues (129) first identified Piezo1 and Piezo2 as mechanically activated ion channels. Piezos are widely expressed in different tissues and organs and are associated with physiological functions such as proprioception and blood pressure (130). Biophysically, Piezo structures have been found to locally deform the cell membrane. Structural studies have determined that Piezo1 adopts a propeller-like triskelion structure with three arms surrounding a central pore (37–40). Remarkably, Piezo1 does not conform to a locally planar membrane and instead locally deforms the cell membrane. One study found that Piezo-related deformations almost always projected into the cytoplasm (Figure 7a,b) (37). They hypothesized that Piezo channel opening flattens the dome-shaped membrane deformation, increasing membrane planarity and consequently increasing the membrane’s projected area. They reasoned that this change in projected area accounted for Piezo1’s sensitivity to changes in membrane tension (37, 131). Numerous structural studies as well as force-related biophysical studies on the curved and flattened structures of Piezo1 have also been conducted, further elucidating the mechanosensitive gating mechanisms of Piezo channels (37–40, 42, 132). The spatial distribution of Piezo1 within a cell is also regulated by membrane curvature. A recent study examined the dynamics and localization of Piezo1 in cells (36). Utilizing nanofabricated substrates and tether pulling assays, the authors demonstrated that Piezo1 is depleted from filopodia with high negative curvature (Figure 7c) and is enriched in membrane invaginations corresponding to positive curvature (Figure 7d).

Transient receptor potential (TRP) channels are another family of ionotropic channels implicated in mechanosensing. Due to the diversity of activation methods, some TRP channels, but not all, are mechanosensitive. Studies in mammalian cells found that TRPV and TRPC localize to areas of positive curvature in cells that are cultured on microtopographical substrates (133, 134). Given TRP’s response to membrane curvature as well as involvement in neurite guidance, Li and colleagues theorized that TRP channels may be involved in neurite alignment to micropatterned substrates, likely by responding to topography-induced membrane curvatures.

### Surface Nanotopography Regulates Cell Migration

Cell migration is involved both in healthy cell functions, like tissue development and regeneration as well as proper immune response and wound healing (135), and in malignant and aberrant cellular behaviors, such as cancer invasion and metastasis (136). While chemotaxis and durotaxis are both highly studied in the field of cell migration, topotaxis (cell movement between surfaces of differing roughness) (137) is a relatively new area of study that is especially relevant in wound healing and cancer cell migration, when cells migrate through different extracellular matrix densities and textures. Though cells migrate in response to chemical and mechanical signals (138, 139), here we focus on the role nanotopography plays in cell migration.

**Figure 8:**
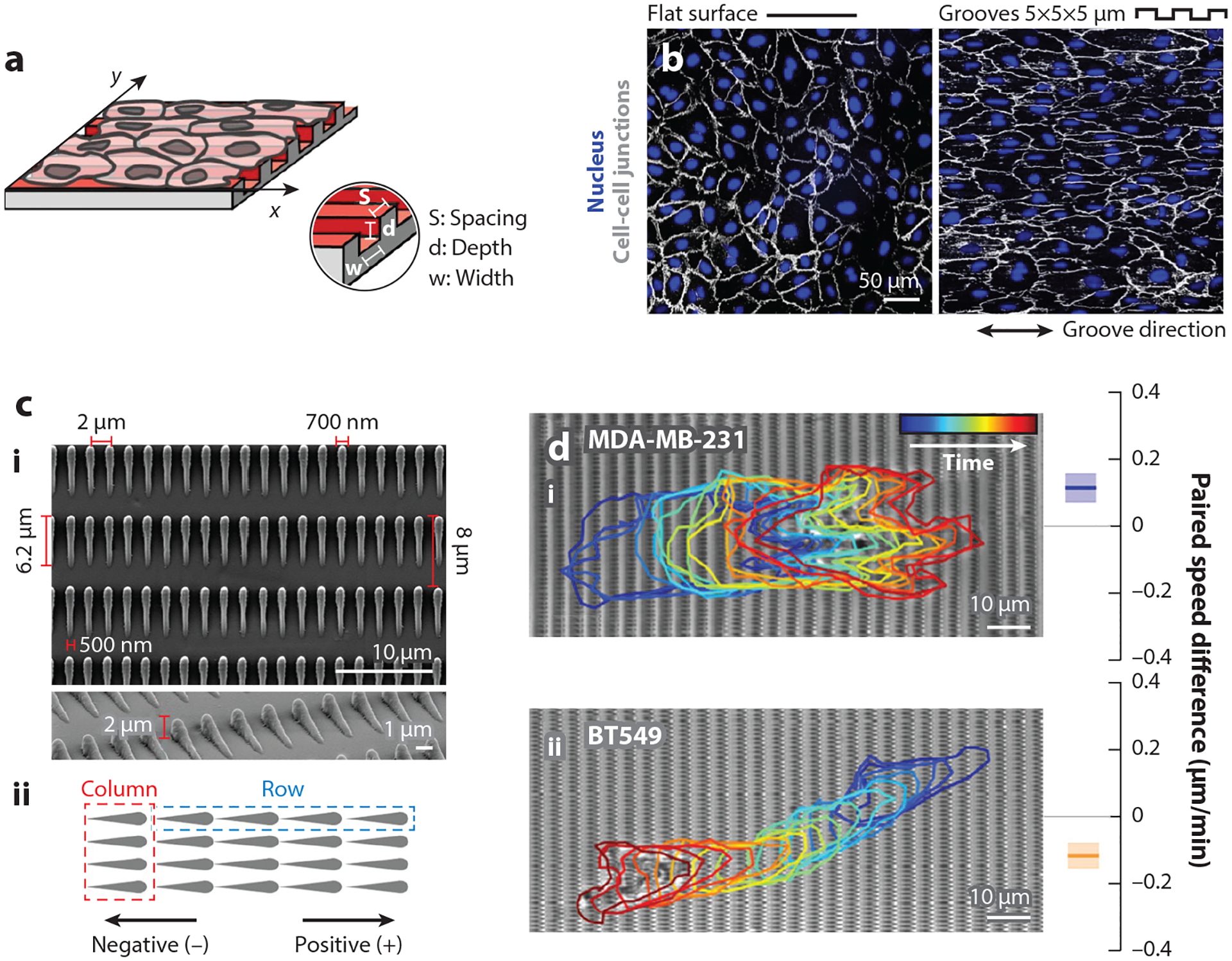
Surface nanotopography modulates cell migration. (*a*) Schematic illustration of an endothelial cell (HUVEC) monolayer on a nanogrooved substrate. (*b*) Representative images of HUVEC monolayer migrating on flat (*left*) or nanogrooved (*right*) substrates. Cell-cell junctions are probed by VE-cadherin immunostaining. Panels *a* and *b* adapted from Reference 142 (CC BY 4.0). (*c*) Scanning electron microscopy images (*i*) and schematic illustration (*ii*) of an asymmetric sawteeth array. (*d*) Bright-field images and paired speed difference analysis of MDA-MB-231 (*i*) and BT549 (*ii*) cells migrating on asymmetric sawteeth substrates. MDA-MB-231 tends to migrate in the positive direction on sawteeth substrates, while BT549 displays the opposite effect. Panels *c* and *d* adapted with permission from Reference 145; copyright 2019 American Chemical Society.

Directional cell migration can be controlled by the orientation of nanotopography. Recently, Cheng and colleagues (140) tested cell migration in grooves by creating microgrooves that were formed using patterns of nanopillars or nanoholes. On nanopillars or nanoholes alone, osteoblast precursor MC3T3 cells move without directional guidance. However, by creating striped patterns of nanoholes and nanopillars, cells move parallel to the direction of the stripes but at a lower velocity than cells migrating on unstriped nanostructured substrates. Another recent work shows that parallel cell alignment along nanowave topography highly depends on cell-cell interactions (141). By quantifying C2C12 cell migration and alignment, it was found that high-density cell seeding is required for cell alignment on nanowave topography. Work by Leclech and colleagues (142) further expands upon this observation by showing that endothelial cell monolayers migrate collectively in antiparallel cell streams when grown on microgroove topography (Figure 8a,b). Interestingly, cell stream movement was much more impacted by groove depth than by groove width. Thus, cell density heavily influences bidirectional cell migration along waves and grooves.

Topotaxis greatly depends on cell type and the pattern of nanotopography. While osteoblast precursor MC3T3 cells move at a higher velocity on nanostructures (143), Lestrell and colleagues (144) found that human neural progenitor cells (hNPCs) have a drastically decreased velocity on silicon nanoneedles. Chen and colleagues (145) found that nanoscale asymmetric sawtooth patterns unidirectionally bias cell movement depending on cell type (Figure 8c). Even though all five cell lines tested were breast cancer cells, distinctive effects were observed in the different lines: MDA-MB-231,HS578T, and MCF10A cells all showed a bias in the direction of the highest point of the sawtooth ridge, while BT549 and MCF10CA1 cells exhibited the opposite effect (Figure 8d). Another study found that two melanoma cancer cell lines, noninvasive SBcl2 and invasive 1205Lu, prefer to migrate toward and accumulate in areas of sparse nanoposts rather than dense ones (146). Recent work by Shivani and colleagues (147) shows that osteosarcoma MG63 cells preferentially migrate to and accumulate in areas of rough nanotextured surfaces rather than smooth nanotextured surfaces. Again, topotaxis appears to be highly dependent on cell type and specific topographical structure, and more studies are needed to further investigate this effect.

### Surface Nanotopography Biases Cell Differentiation

Cell differentiation is a complex process controlled by a myriad of factors, such as growth factors, chemical stimuli, extracellular matrix factors, and other microenvironmental elements (148). It is well established that nanotopography plays a major role in modulating stem cell differentiation (149, 150). Optimization of nanotopographical features can predictably and specifically guide cell differentiation into different cell types, including osteoblastic, myoblastic, chondrogenic, fibroblastic, and neural cells.

**Figure 9:**
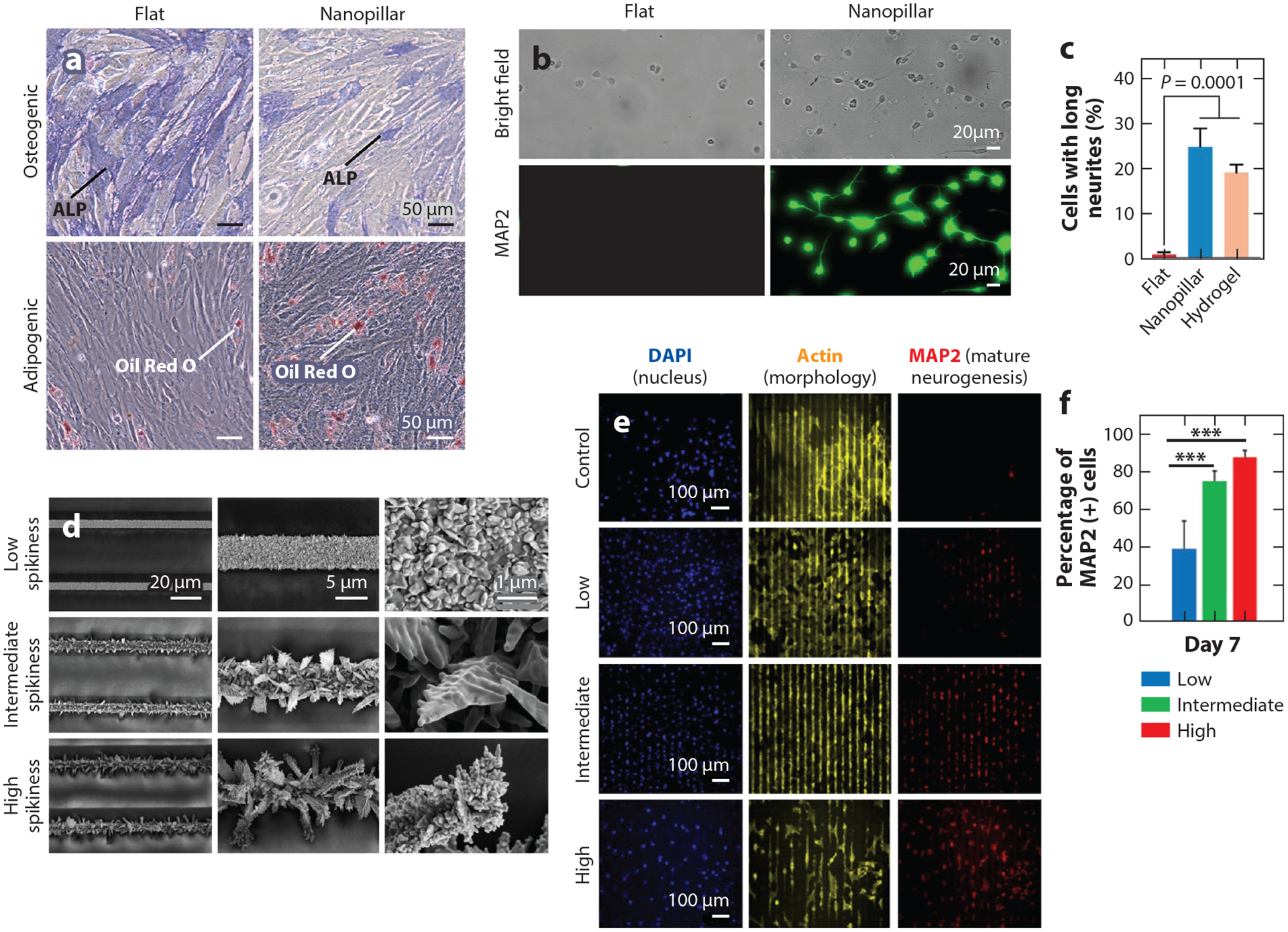
Surface nanotopography biases cell differentiation. (*a*) Bright-field images of hMSCs grown on flat (*left*) or nanopillar (*right*) substrates. ALP serves as a marker for osteogenic differentiation, while Oil Red O is used as an adipogenic differentiation marker. hMSCs preferentially differentiate into adipogenic fat cells over osteogenic bone cells when growing on nanopillar substrates. In contrast, they preferentially undergo osteogenic differentiation when growing on flat surfaces. (*b*) Bright-field (*top row*) and fluorescence (probed by MAP2 immunostaining) images (*bottom row*) of E18 rat hippocampal neurons cultured on the flat area (*left column*) or the nanopillar area (*right column*) of a quartz substrate. Hippocampal neurons form long neurites when growing on nanopillar areas. (*c*) Quantifications of percentage of hippocampal neurons forming long neurites on three different substrates. Panels *a–c* adapted with permission from Reference 63; copyright 2021 American Chemical Society. (*d*) Scanning electron microscopy images of microgrooved substrates patterned with nanotextures of different spikiness. (*e*) Fluorescence images of hMSCs cultured on microgrooved substrates with different nanoscale spikiness. Control denotes the microgrooved substrate possessing little or no nanotextures. MAP2 is used as a marker for mature neurogenesis. A significant number of hMSCs undergo neurogenesis when growing on highly or intermediately spiky substrates. (*f*) Quantifications of percentage of MAP2-positive hMSCs cultured on microgrooved substrates with different nanoscale spikiness. Panels *d–f* adapted with permission from Reference 156; copyright 2018 American Chemical Society. Abbreviations: ALP, alkaline phosphatase; hMSC, human mesenchymal stem cell; MAP2, microtubule-associated protein-2.

The balance between osteoblast and osteoclast differentiation is topographically dependent. Li and colleagues (151) found that titanium surfaces roughened to a nanotexture promoted osteogenic differentiation in vivo compared to macrotextured and smooth surfaces. A 1.8- to 2-fold increase in osteogenic differentiation was also observed when titanium implants were modified with nanodome (50 nm in diameter, 25 nm in height) topography (152). In contrast, when human mesenchymal stem cells are grown on high-aspect-ratio (200 nm in diameter, 1–3 μm in height) nanopillars, cells preferentially differentiate into adipogenic fat cells over osteogenic bone cells (Figure 9a) (63). Another recent study showed that larger, relatively low-aspect-ratio nanopillars (500 nm to 1 μm in diameter, 500 nm in height) promoted osteoclast formation, yet smaller, relatively high-aspect-ratio ones (100 nm in diameter, 200 nm in height) reduced osteoclast formation (153). Nanotopography has even been reported to inhibit mesenchymal stem cell differentiation. Nanopits can be used to maintain the mesenchymal stem cell phenotype while still allowing for cell growth (154, 155). Thus, topography-induced cell differentiation is greatly dependent on topography shape, size, and height.

Regulation of neuronal differentiation is also highly desired in the context of studying neuronal damage and neurodegenerative diseases. Li and colleagues (63) observed markedly increased neurite growth when neurons were grown on nanopillar surfaces or soft hydrogels as opposed to flat surfaces (Figure 9b,c). Poudineh and colleagues (156) showed that microgrooves decorated with highly spiky nanotextures promote neurogenic differentiation of human mesenchymal stem cells (Figure 9d). This process is highly efficient, as over 80% of cells express mature neuronal marker MAP2 (microtubule-associated protein 2) after a week of culture on spiky nanotextures (Figure 9e,f) (156). Thus, nanotopography imparts a driving force in neurogenic differentiation.

## CONCLUDING REMARKS

The growing body of research on membrane curvature demonstrates that membrane curvature is inextricably linked with cellular processes. The scope of work on membrane curvature is extensive. Previous studies have ranged from the identification of curvature-sensing and membrane-shaping lipids and proteins to the observation of the physiological relevance of membrane curvature. Despite the breadth of membrane curvature research, numerous open questions remain: Of the known mechanisms to change membrane curvature, what are the mechanical forces and free energies at play to change membrane shape? There are multiple curvature-generating mechanisms, but known mechanisms fail to explain the behavior of certain curvature-associated proteins (157): What are other curvature-generating mechanisms? Are there unidentified curvature-sensing or curvature-generating proteins? What are the specific roles of individual proteins in curvature assemblies? Certain curvature mechanisms have been theorized but have not yet been experimentally determined (24) and vice versa: How can we bridge the gap between theory and experimental evidence for membrane curvature? Lastly, the significance of membrane curvature has been experimentally shown in vitro and in cellular experiments: What is the physiological role of membrane curvature in vivo?

Future advances in manipulating the membrane as well as in capturing membrane curvature dynamics will be necessary to better understand the physiological and pathological roles of membrane curvature. For instance, since the scale of membrane curvature pushes the resolution limit, the development of faster, high-resolution microscopy methods will be necessary to capture the spatial conformation and dynamics of curvature-sensing proteins. Furthermore, although we have highlighted some methods to dynamically control membrane curvature, new methods that can manipulate membrane curvature in real time will be crucial. We anticipate that the development of new methods and continued research on membrane curvature across numerous fields, including physical chemistry, biochemistry, cell biology, and others, will lead to new, compelling discoveries in the field of membrane curvature.

## Figures and Tables

**Table 1:** Summary of membrane curvature-sensing and curvature-generating mechanisms and related proteins

Mechanism	Examples	Curvature-sensing mechanism	Curvature-generating mechanism	Reference(s)
Geometric match between proteins and curved cell membranes (Figure 1b)	BAR-family proteins (Figure 1b, subpanel *i*)	Crescent-shaped, cationic BAR domains sense and bind to anionic curved membranes. In general, N-BAR (e.g., amphiphysins, endophilins) and F-BAR (e.g.,FBP17, FCHo2) proteins sense positive curvature; I-BAR (e.g., IRSp53) proteins sense positive curvature.	Oligomerization of BAR-family proteins scaffold cell membranes. In general, N-BAR and F-BAR proteins induce membrane invaginations; I-BAR proteins induce membrane protrusions.	(27–31)
	Wedged/conical transmembrane proteins (Figure 1b, subpanel *ii*)	Preferential partition into the curved segments of cell membranes with matching intrinsic curvature. For example, KvAP, KvChim, and Piezos prefer positive curvature; GPCRs prefer negative curvature.	Clustering of wedged/conical transmembrane proteins sculpt cell membranes with matching curvature. Clustering of KvChim or Piezos generates positive curvature.	(32–35, 37–40, 42)
Insertion of AHs into lipid packing defects within curved cell membranes (Figure 1c)	ALPS motif–containing proteins	Insertion of AHs into the accessible lipid packing defects within curved membranes, mostly with positive curvature. Examples of ALPS motif–containing proteins are ArfGAP1, coat protein complex (e.g., COPI/II), α-synuclein, ENTH-family proteins (e.g., epsin 1/2/3/4), EHD-family proteins (e.g., EHD 1/2/3/4), and several N-BAR (e.g., amphiphysins, endophilins).	Clustering of AHs within the inner leaflets mostly induces positive membrane curvature.	(45–48)
Molecular crowding of membrane-bound proteins (Figure 1d)	Membrane-bound bulky structured proteins (Figure 1d, subpanel *i*)	Steric effect: preferential partition into curved membranes with convex curvature, through which to reduce intermolecular steric repulsions and increase configurational entropy. Electrostatic effect: preferential partition into curved membranes with convex curvature, through which to reduce intermolecular electrostatic repulsions and increase configurational entropy. Examples of membrane-bound bulky structured proteins and/or polymer-like IDPs are MUC1 and podocalyxin (with anionic, bulky extracellular N-terminal IDRs), hyaluronic acid (with anionic, bulky extracellular IDRs), AP180 (with anionic, bulky intracellular C-terminal IDRs), and epsins (with bulky intracellular C-terminal IDRs).	Steric effect: At high surface density, membrane-bound bulky structured proteins or IDPs exert steric pressures to curve cell membranes toward them to reduce free energy. Electrostatic effect: At high surface density, membrane-bound anionic IDPs bend cell membranes toward them to reduce free energy. Depending on the location (extracellular or intracellular) and orientation of membrane tethers, the convex or concave curvature they sense and induce can be either a positive or a negative membrane curvature.	(53)
	Membrane-bound polymer-like IDPs (Figure 1d, subpanel *ii*)			(49, 50, 158)
	Membrane-bound proteins with strong intermolecular interactions (Figure 1d, subpanel *iii*)	Preferential partition into curved membranes with concave curvature, through which to maximize protein-protein interactions and minimize free energy. Examples of membrane-bound proteins with strong intermolecular interactions are FUS and hnRNPA2.	At high concentrations, proteins oligomerize into biomolecular condensates, which push membrane bilayers outward, resulting in concave curvature.	(54, 55)

Abbreviations: AH, amphipathic helix; ALPS, amphipathic lipid packing sensor; EHD, Eps15 homology domain; ENTH, epsin N-terminal homology; GPCR, G protein-coupled receptor; hnRNPA2, heterogeneous nuclear ribonucleoprotein A2; IDP, intrinsically disordered protein; IDR, intrinsically disordered region.
